# Updates in the Diagnosis and Treatment of BK Viraemia in Kidney Transplant Recipients: Current and Future Insights

**DOI:** 10.3390/jcm14217759

**Published:** 2025-11-01

**Authors:** Donnchadh Reidy, Dearbhail Ni Cathain, Sam Kant

**Affiliations:** 1 Department of Nephrology, St. Vincent’s University Hospital, D04 T6F4 Dublin, Ireland; donnchadhreidy@svhg.ie (D.R.); dearbhailnicathain@alumnircsi.com (D.N.C.); 2 Department of Nephrology, St. Vincent’s University Hospital, University College Dublin, D04 T6F4 Dublin, Ireland

**Keywords:** BK virus, kidney transplantation, kidney injury, nephropathy, viraemia

## Abstract

BK virus is a common childhood infection that is largely asymptomatic in the general population. However, increased cellular immune dysfunction in kidney transplant recipients is associated with an increased risk of BK virus reactivation. Modern immunosuppression regimens have resulted in a reduction in transplant rejection events but increased risk of BK nephropathy. It is now considered a leading cause of allograft loss within the first year of transplantation. Despite advances in screening, it remains both a diagnostic and therapeutic challenge. This review aims to provide an up-to-date summary of the latest clinical research in the diagnosis and treatment of BK virus in kidney transplant recipients. It will also provide a concise overview of emerging diagnostic techniques and new therapies under investigation.

## 1. Introduction

BK virus was initially isolated in the urine of a 39-year-old Sudanese kidney transplant recipient with the initials B.K. in 1971, who presented with ureteric obstruction from a ureteral stricture [1]. BK nephropathy was subsequently diagnosed on biopsy in 1993. The incidence of BK virus has been increasing since its initial discovery in 1971. This is likely secondary to increasing availability of testing methods and modern potent immunosuppressive regimens. It is now recognised as an important cause of allograft loss particularly within the first year following transplantation. For this reason, it is considered an area of considerable interest for emerging research in transplant medicine. This review provides an up-to-date summary of the latest available published clinical research on BK virus on diagnosis, treatment and emerging therapies.

## 2. Viral Structure and Mechanism

The BKPyV genome is a closed circular double-stranded DNA molecule of approximately 5 kb that replicates bidirectionally from a unique origin. BK virus is a betapolyomavirus genus of the polyomaviradae which also includes simian virus 40 and JC virus. Its sequence has a 75% and 69% genetic similarity with JC virus and SV40, respectively. The genome of BK virus has an early region which codes for the large and small T antigens, a late region which codes for the capsid proteins VP1-3, and agnoprotein, and a non-coding control region. BK virus has six genotypes based on polymorphisms in VP1 and the non-coding control region (NCCR) [2]. Subtype I is the most common worldwide and is typically implicated in the most clinically significant viral diseases followed by Subtype IV [3]. Its capsid is icosahedral in structure and non-enveloped with a diameter of 40 to 44 nm comprised of the encoded capsid proteins VP1, VP2 and VP3. The capsid proteins are arranged in an icosahedral structure containing VP1 organised in 72 pentamers with each pentamer linked internally to a single capsid protein; VP2 or VP3 [4]. The main function and structure of the BKPyV components are listed below in Table 1.

Further work has been done furthering our understanding of the mechanism of BKPyV replication. The current model is that BKPyV expresses the large T antigen (TAg) early during the infection, promoting cells to enter S phase where the viral DNA can access host replication machinery. However, a recent single-cell analysis that involved inhibiting host DNA replication and not viral replication demonstrated that TAg expression and viral production rely on an initial host S phase, and that BKPyV primarily replicates during host re-replication [15]. Importantly, BK virus infection does not always result in BKPyV nephropathy; in most cases, the viral replication is controlled by the immune system. It has been shown that patients who have higher and broader antibody reactivity at transplantation are more likely to be able to clear BK virus at 6 months and 12 months post-transplant [16].

Notably, variance amongst individual HLA antigens may also lead to some explanation as to varying responses to BK viraemia; however, outcomes are complex and remain poorly understood [17]. Recently it was demonstrated that HLA-DR T-effector cell counts at day 10 post-transplant are associated with increased tacrolimus therapy and rates of BK viraemia when adjusted for relevant confounders [18]. This could support the use of HLA-DR T-effector cell counts as a biological marker of calcineurin inhibitor effect, and thus risk of BK viraemia. Knowledge of the effect of HLA variants on T cell response is also significant when selecting donors for virus-specific therapy (VST). A large registry of VST donors has demonstrated the complexity of T cell response depending on individual HLA alleles [19].

## 3. Epidemiology

BK virus tends to occur in early childhood with increasing prevalence with age. A Finnish study demonstrated that 98% of children had antibodies to BK virus by the age of 9 [20]. Whilst the exact route of transmission of BK virus is unknown, it is thought likely that it is via the respiratory tract or faecal–oral route [21,22]. It may also be transmitted via semen, urine, blood transfusion or organ transplantation [23]. Studies in the adult population have estimated the prevalence of BK antibodies to be about 80% [24,25]. Detectable antibody prevalence declines by almost 9% for every 10 years of age [26].

Clinically significant infection typically occurs within the first year post-transplantation as this is when cellular immunity is at its lowest due to induction therapy. Following transplantation, infection likely occurs due to reactivation of latent infection or transmission of new infection from the donor kidney. Infection has been initially postulated to result in viruria progressing to viraemia and then transplant nephropathy [14]. Within the renal transplant cohort, BK viruria can be detected in up to 30% of patients [27]. BK viraemia occurs in 10–20% whilst BK-associated nephropathy occurs in approximately 3% of patients [28,29,30]. Despite the serious complications of BKPyV DNAemia, including BKPyV nephropathy which was largely irreversible until recently, there have been few prospective studies examining the natural history of BK virus in kidney transplant recipients. A recent multi-centre prospective study in the US performed longitudinal surveillance on kidney transplant and simultaneous pancreas–kidney transplant patients where patients were screened every 4 weeks for BK viraemia [31]. Those with PCR-confirmed BK viraemia were monitored every 2 weeks. This study suggested that male donor sex was associated with lower odds for BK viraemia. Recipient black ethnicity was associated with two-fold higher odds of BKPyV DNAemia.

Risk factors can be broadly categorised into donor risk factors, recipient risk factors and transplantation factors. Risk factors for BKPyV DNAemia and BK nephropathy are outlined in Table 2. Event rates in the available literature are much higher for BKPyV DNAemia, given the prevalence of testing providing a higher evidence profile [32].

It has long been recognised that rates of BKPyV nephropathy have been significantly higher in the ABOi transplant population. It was unclear whether this was an intrinsic attribute of the ABOi transplants or as a result of the associated increased immunosuppression. A recent study using Torque Teno virus demonstrated that ABOi transplants have greater tacrolimus exposure and immunosuppression, with significantly greater levels of BKPyV nephropathy 24 months post-transplant when compared with HLA-incompatible transplants [33]. It was suggested the increased BKPyV nephropathy rates are due to the immunosuppressive burden rather than any intrinsic attribute of a ABOi transplant. The standard treatment for BKPyV DNAemia is a reduction in immunosuppression; however, this is not risk-free and must be weighed up against the risk of alloimmune response. A retrospective analysis of 460 kidney transplant patients demonstrated that BK virus status within the first 6 months of transplantation can be a predictor for future T cell-mediated rejection (TCMR), particularly in patients with greater underlying molecular mismatch [34].

A recent systematic review of potential modifiable risk factors for the development of BK virus-associated complications highlighted the complexity of studying risk factors, given the possibility of confounding. No risk factor identified significantly affected all of the end-points. However, greater immunosuppression with corticosteroids, tacrolimus and use of anti-thymocyte globulin were seen across multiple end-points [35].

## 4. Clinical Presentation

BK virus is a common infection in childhood which is largely asymptomatic and does not result in major clinical sequelae [2]. During infection, BKPyV can be disseminated by peripheral monocular cells to the urinary tract where it enters a non-replicative phase in the renal tubular epithelial cells of urothelium [2,36]. This can later manifest as periodic asymptomatic urinary shedding of the virus in healthy adults due to reactivation of BKPyV [37].

BKPyV has a more significant role in the immunosuppressed cohort, particularly following organ transplantation. BK-associated nephropathy (BKVAN) progresses to graft failure in up to 40% of BK virus-associated nephropathy cases [38,39]. BK-associated nephropathy typically occurs within the first year of transplantation due to reduced cellular immunity following induction therapy. One study examining a cohort from Australia and New Zealand reported a median time to disease occurrence of 4.8 months post-transplant [28]. Another potential sequelae of BK infection includes ureteral stenosis, although the magnitude of its effect remains relatively unknown [40]. Whilst uncommon in the renal transplant population, haemorrhagic cystitis is a well-recognised complication of BK virus in patients post-bone-marrow-transplant [41].

There are multiple published reports of BK virus infection of rodent models or cells in culture resulting in tumour formation or transformation [42]. As BK virus sustains a persistent latent stage in epithelial cells of renal tubules or urothelium it has been speculated that there is increased oncogenesis of urothelial tumours particularly in the transplant cohort [14,43,44]. Despite increasing reports linking BK virus to human malignancy, a causative role in humans remains controversial. This is due to conflicting results regarding the presence of BK virus sequences and proteins in various tumour types [14,45]. Much of the available research is affected by confounding, as patients who have higher levels of BK virus and BK nephropathy have decreased cellular immunity, and thus are at increased risk of tumorigenesis.

## 5. Diagnosis and Histology

**Figure 1:**
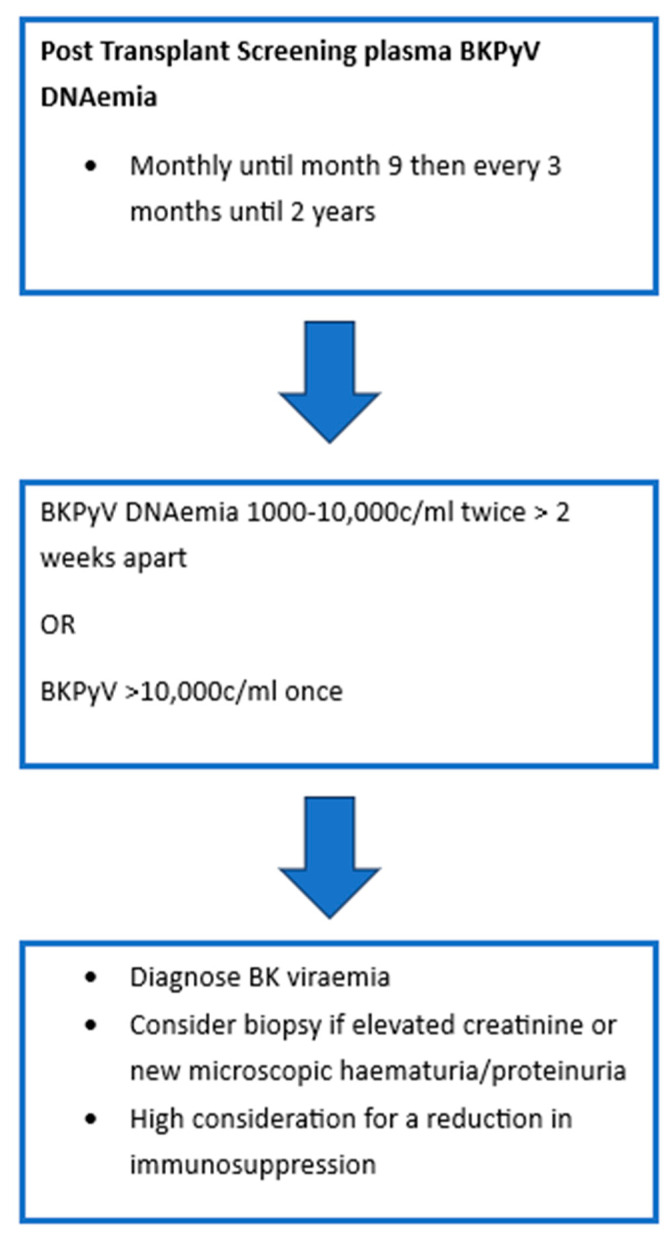
BKPyV Screening Protocol.

As BKPyV infection or reactivation post-transplant is largely asymptomatic, most programs have adopted screening protocols. Figure 1 demonstrates the most recent International Consensus Guidelines on the Management of BK Polyoma Virus in Kidney Transplant recommendations for screening [32]. In patients with sustained levels > 1000 c/mL, monitoring of BKVyP DNAemia is recommended every 2–4 weeks to monitor for response to intervention. Should patients require increased levels of immunosuppression then they should have monthly monitoring of BK viral levels for 3 months. BKPyV DNAemia is considered to be two to three consecutive measurements > 1000 c/mL or one single measurement > 10,000 c/ml [32,46]. A recent Cochrane review of urinary BKPyV QNAT compared its efficacy to that of serum and demonstrated that there was insufficient evidence to suggest the use of urinary testing as a primary screening tool. Serum testing with a cut-off of 10,000 copies/mL was robust with a sensitivity of 0.86 and specificity of 0.95. Of note, it was suggested that a lower cut-off closer to 2000 copies/mL may be more optimal as it would increase sensitivity to 0.89. However, it was acknowledged that the current cut-off of 10,000 copies/mL did offer good performance characteristics and supported the current recommendations [47].

Kidney biopsy remains the gold standard method for diagnosis of BKPyV nephropathy however, it may still fail to detect intra-graft replication in 10–30% of cases during early onset or when biopsy proven nephropathy is resolvingresolving [32,48,49,50,51]. Current guidelines do not advocate for routine biopsy of every patient with BKPyV DNAemia; however, selecting those with high immunological/virologic risk or those with rising creatinine, haematuria and/or proteinuria is advisable [32]. Terminology in the field of BK nephropathy refers to ‘presumptive’ BK nephropathy in those with high viraemia titres without histological confirmation of disease versus ‘definitive’ BK nephropathy which is biopsy-proven [52]. A definitive diagnosis of BK nephropathy requires the presence of the characteristic cytopathic changes and positive immunohistochemistry staining [53]. Given the limitations of nucleic acid testing in the screening of BK virus, it has been speculated that protocolised biopsies may allow clinicians to detect BKPyVAN at an earlier stage, thus improving reversibility. A large cohort of over 300 protocolised biopsies detected BKPyVAN in 3.8% of their cohort with the majority at an early stage. All patients maintained their graft function on reduction in immunosuppression with a median follow-up of 6 years [54]. Protocolised biopsy may offer earlier detection; however, given the low rates of BKPyVAN, the risk of biopsy must also be considered.

Light microscopy demonstrates a patchy interstitial infiltrate made up of lymphocytes, plasma cells and some neutrophils and usually demonstrates co-existent interstitial oedema, tubulitis and tubular injury [53]. Due to the affinity the BK virus has for urothelium and renal tubular cells, viral inclusions are most often seen in the medulla and distal tubule, but can progress to involve the proximal tubule and the parietal epithelium [14]. The infected cells are seen to have enlarged nuclei with amorphous inclusions. The inclusions are described as ground-glass with irregular central clearing or as granular inclusions with clumping of intranuclear viral material [14,53]. BK nephropathy can demonstrate subtle, focal changes that are limited to the medulla, so it is recommended that two cores are sought with both cortex and medulla in order to optimise the chance of capturing the above detailed viral inclusions [52,55].

Immunohistochemistry is another useful tool that can capture BK nephropathy at early stages of the disease process [56]. The simian virus 40 (SV40) stain is non-specific to BK virus, but is instead a broader marker of polyoma virus infection, staining the large T antigen expressed by all polyoma viruses [52,53,57]. Immunofluorescence can show staining for IgG, C3 and C4d, usually in a granular distribution along the tubular basement membrane [53]. C4d staining is standard practice in the analysis of renal transplant biopsies with peritubular capillary staining aiding diagnosis of antibody-mediated rejection. However, there is emerging evidence that C4d staining along the tubular basement membrane is highly prevalent in BK nephropathy, with diffuse C4d staining associated with poorer transplant outcomes and potentially useful as an independent risk factor for graft loss [58,59,60].

Electron microscopy can demonstrate classical polyomavirus particles and intranuclear inclusions, usually 30–45 nm, that can be arranged in a lattice-like pattern [53].

The Banff Working Group on Polyomavirus Nephropathy (PVN) worked to create a classification system that combined assessment of polyomavirus replication or viral load and the extent of interstitial fibrosis to provide a more standardised approach to diagnosis and prognosis and to aid research definitions in this area [61,62]. They defined three distinct classes of PVN (Table 3) and proposed that the classes were predictive of clinical presentation at the time of diagnosis, renal function during follow-up and graft loss, with class 3 conferring the worst prognosis [52]. Subsequent studies have validated the clinical utility of this morphological classification [61].

There are many challenges with the current screening and diagnostic methods outlined above, including concerns regarding missed/late diagnoses and difficulty distinguishing T cell-mediated rejection on biopsy. For this reason, multiple new methods for screening and diagnosis are in development. Below is a list of emerging diagnostic tools that are currently in research:

1. Urinary-derived mitochondrial cell-free DNA was utilised to aid in the diagnosis of BKPyVAN [63]. Mitochondrial dysfunction is often seen in BK-associated nephropathy. In a cohort of 60 kidney transplant recipients, it was shown that the ratio of urinary-derived cell-free mitochondrial DNA to donor-derived cell-free DNA had a sensitivity of 71.4% and a specificity of 97.1% [63]. Given the increasing use of donor-derived cell-free DNA in clinical practice this may be a useful adjunct in guiding clinicians in adjusting immunosuppression whilst also avoiding the risk of an invasive procedure such as a biopsy [64,65]. However, given the small cohort, further studies will be needed to validate this method.
2. Urine lateral flow assays are another tool under consideration as they may offer a more cost-effective solution than quantitative nucleic acid testing and be of use in a paediatric cohort where blood sampling may be more challenging. Current limitations of BK virus QNAT testing include under-quantification and false negatives. It has been well-established that high-level BK viraemia precedes DNAemia making it a potentially useful biological source to screen for early BK virus activity [66]. A urine lateral flow test for BKPyV VP1 antigen has been developed and shown to be effective in vitro [67]. However, further multi-centre data is awaited to elicit if it will be beneficial in clinical practice.
3. Urinary micro-RNA is another tool under consideration to improve the accuracy of screening for early evidence of BK virus. Two micro RNAs of interest were examined in a small cohort of kidney transplant recipients in Japan. Both demonstrated value as a screening tool; however, further research is required in larger cohorts prior to clinical application [68].
4. Single-cell DNA studies aim to define the unique molecular signature of BK nephropathy to improve the understanding of the pathogenesis and to allow for the development of future diagnostic methods. A recent single-cell study demonstrated the unique cell populations and dynamics between T cell-mediated rejection and BK nephropathy [69]. It demonstrated a predominant rise in T cells with the main cell differences occurring in the proximal tubule, principal cells and thick ascending limb as well as the medulla in BK nephropathy compared to T cell-mediated rejection. Whilst this is unlikely to be used as a diagnostic method, it offers further targets for research into new methodology.
5. In situ hybridisation (ISH) targeting nucleic acids is of particular interest for kidney biopsy specimens as it may offer improved diagnostic specificity over established methods. Established methods for kidney biopsy assessment can mean that it is difficult to distinguish rejection in certain cases and there may be overlap with SV40-positive viruses. A research group in China utilised a ViewRNA ISH system to detect the conserved sequences within the large T cell antigen of BK virus to improve diagnostic accuracy. They demonstrated a 93.75% accuracy using this model [70].
6. Risk prediction models would offer better stratification of at-risk transplant recipients and may allow for a more tailored approach to screening. Despite the well-established risk factors for BKPyVAN outlined above, risk prediction remains an ongoing challenge, as does the impact of various risk factors. Attempts have been made to develop risk prediction models; however, their predictability is limited. One recent study developed a three-item model performed at the time of transplant with a scoring system ranging from 0–4 based on factors including age, sex and previous kidney transplantation [71]. Notably, it did have some predictive value, but its area under the curve was only 0.66 at one-year post-transplant.

## 6. Treatment

It has previously been demonstrated that persistent BKPyV DNAemia is a dominant driver for allograft injury and that delays in viral clearance (detectable at >6 months) are associated with an increased risk of graft failure [72]. The goal of treatment is to lead to a sustained reduction in BKPyV DNAemia to undetectable levels. In the absence of an effective anti-viral therapy in BK nephropathy, the main therapeutic intervention is the reduction in maintenance immunosuppression [48]. Historically, treatment strategies have been centre dependent and highly variable, depending on local protocols without supporting randomised control trials [48]. Practices can vary significantly across the lifespan of the transplant and can include induction therapy, maintenance immunosuppression and screening protocols for BKVAN, even before management strategies are examined. This creates a number of challenges and confounders before treatment strategies are even assessed [55]. The “Second International Consensus Guideline on BK Polyomavirus in Kidney Transplantation” combined multinational expert opinion to review the current best evidence and establish a standardised flow chart for the screening, diagnosis and management of BKVAN, and established graded recommendations [32]. Based on this consensus the following was established:

Reduction in immunosuppression is advised in those with the following:

1. Confirmed BK viraemia demonstrated by titres of 1000–10,000 copies/mL on two measurements 2–3 weeks apart.
2. Confirmed BK viraemia demonstrated by titres of >10,000 copies/mL on one measurement.
3. Biopsy-proven BK nephropathy.

**Figure 2:**
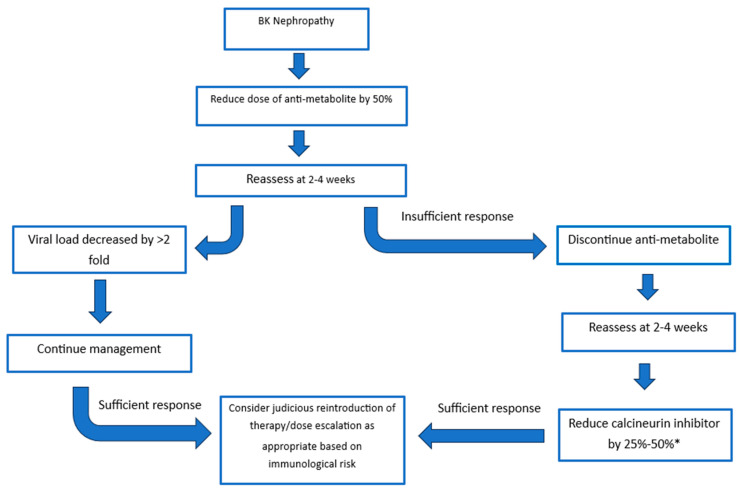
Treatment Strategies. A sufficient response is a decrease in viral load >2 fold. * Aim for tacrolimus trough of 3–5 ng/mL or a ciclosporine trough of 75–125 ng/mL.

The recommendations of the consensus group were built on the guidelines released in 2019 by the American Society of Transplantation [48]. Treatment strategies consider the initial reduction in the anti-metabolite versus initial reduction in the calcineurin inhibitor. The response to the dose reduction is monitored at 4 weeks with options to further reduce or discontinue agents until an adequate response is achieved (Figure 2) [32]. There is evidence to support reducing the anti-metabolite or calcineurin inhibitor. Studies evaluating the initial reduction in anti-metabolite therapy have mainly focused on the use of mycophenolate mofetil (MMF) and have found dose reduction and cessation of MMF to be a safe and effective method of clearing BK viraemia, and this has supporting 5- and 10-year follow-up [30,32,73,74]. The risk of acute rejection was not significantly increased once patients were maintained on both steroid and CNI therapy [30]. A “CNI-first” approach has substantial supporting evidence with retrospective analysis demonstrating similar transplant outcomes at 6.6 years in those with and without BK viraemia [75]. All immunosuppression reduction strategies must be weighed up with the risk of rejection. A recent multi-centre analysis of immunosuppressive strategies demonstrated that tacrolimus levels below 5 ng/mL, and complete withdrawal of calcineurin inhibitors significantly increased rejection risk [76]. In the same study a tacrolimus level of 5 to 7 ng/mL led to optimal viral control, whilst balancing rejection rates. Of note, sirolimus-based strategies led to the highest risk of rejection with patients experiencing a near 6-fold higher rate of rejection compared to the mycophenolate control group [76].

Belatacept is being increasingly used in transplant immunosuppression in those with CNI nephrotoxicity with favourable long-term outcomes in terms of graft survival, but there has been no appreciable difference in risk of BK DNAemia [77,78].

The consensus group advised against the use of cidofovir, leflunomide, fluoroquinolones and statins for the prevention and treatment of BK nephropathy due to lack of supporting evidence in their favour [32,55,79,80,81]. Recently published phase I/II trial data for cidofovir in BK nephropathy has demonstrated that while it is safe, low-dose cidofovir has no specific BK anti-viral effects and had no significant effect on BK-associated nephropathy [82]. Pulsed intravenous methylprednisolone used for 3 days at diagnosis of BK nephropathy versus continuation of prednisolone 5 mg was not associated with significant difference in outcome measures [83]. The only adjunctive therapy supported in the guidelines is the administration of IVIG for improved BK viraemia clearance and to reduce the risk of rejection in those requiring significant immunosuppression reduction to clear the BK virus [32,55]. IVIG appears to be a safe, well-tolerated therapy with single-centre studies showing improved viral clearance and in vitro models suggesting that early, prophylactic administration of IVIG reduces the spread of infection and may prevent BK-associated complications [84,85,86].

It had been suggested that the use of mTOR inhibitors was associated with a lower incidence of BKN [87]. This was the basis for the recent BKEVER study, a muti-centre, randomised controlled trial evaluating the impact of MMF replacement by Everolimus (EVR) versus standard dose reduction in MMF [88]. Unfortunately, there was no expedient clearance of BK viraemia in the EVR group [55,88].

Adjunctive therapies:

- IVIg may be used with an inappropriate response to immunosuppression reduction to facilitate viral clearance or to prevent rejection in those with a high immunological risk requiring immunosuppression reduction for clearance.
- In steroid free regimens, the addition of steroids should be considered to avoid CNI monotherapy

## 7. Re-Transplant

Allograft loss due to BK nephropathy is not a contra-indication to repeat transplant and in fact, re-transplant in this population appears to have favourable outcomes [89,90]. Ideally, BK viral load should be undetectable prior to subsequent transplant [90] but persistent detectable levels is not an absolute contra-indication to proceeding to transplant [91,92]. Allograft nephrectomy is not recommended as standard in those with persistent viraemia or prior to retransplant as there is no evidence that this intervention prevents future infection and may increase the risk of sensitization [14,93,94].

## 8. Emerging Therapies

BK nephropathy is an expanding area of research with multiple ongoing clinical trials with the hopes of establishing a therapeutic drug target. Potential targets or new therapies under investigation are summarised below in Table 4:

### 8.1. Cellular Immunotherapy

1. Viral-specific T cell therapy (VST) seeks to selectively increase the BK virus T cell subset without compromising overall immunosuppression [95]. One product currently in research is Posoleucel which is an off-the-shelf allogeneic, multivirus-specific T cell therapy [96]. It is manufactured from peripheral blood mononuclear cells obtained from healthy donors with confirmed seropositivity to BK virus, CMV, adenovirus or Epstein–Barr virus, as well as human herpes virus 6. It was initially of interest in the treatment of cytomegalovirus, but has since been used in several clinical trials evaluating it as a treatment option in BK infection particularly in stem-cell transplant recipients and now in solid-organ transplant recipients. Adverse events have been reported with this therapy in previous trials including cytokine release syndrome and graft-versus-host disease. It is also a costly, labour-intensive therapy, and studies have yet to establish the necessary cell type and infusion frequency for the best therapeutic response [55,81,96,97,98,99]. Alternative VST treatments with different methods of manufacturing have demonstrated safety in kidney transplant patients in initial clinical trials [100]. VST has been used to treat patients with haemorrhagic cystitis, BKPyV viraemia and nephritis, with the majority of patients having a complete or partial response to therapy. VST is a promising therapy requiring further research [95,99].
2. Extracorporeal photophoresis (ECP) has been used clinically since 1988 in the treatment of cutaneous T cell lymphoma. It has evolved as a therapeutic strategy used in solid-organ transplantation in the treatment of acute and chronic graft rejection, mainly in the field of lung and cardiac transplantation [101]. ECP is an immunomodulatory therapy that involves the infusion of autologous cellular products obtained via leukaphoresis and exposure to ultraviolet light [102]. Initially, it was thought its immunomodulating benefits were from apoptosis of leukocytes. However, it is likely more complex, involving initiation of tolerogenic dendritic cells that phagocytise apoptotic cells, release soluble factors, and promote regulatory T cell responses and anti-inflammatory mediators, thereby reducing proinflammatory cytokines [101]. ECP is a favourable treatment of rejection as it appears efficacious and does not increase the risk of infection [103]. ECP has been used in case reports as a successful therapy that allowed for immunosuppression minimisation to treat BK nephropathy whilst limiting the risk of rejection [104]. Given the lack of guidelines for ECP in kidney transplantation, and without an effective strategy for patient selection for treatment, it is unlikely to be a standard therapy for BK nephropathy in the near future. However, there is significant interest in developing the research behind this therapy to eventually conduct a multi-centre study [102].

### 8.2. Antibody-Based Therapy

There is strong evidence that a high BKPyV-neutralising antibody titre correlates with a lower risk of developing BKPyV DNAemia following transplant [105]. There are two human-derived immunoglobulin G-neutralising antibodies against VP1 under investigation. VP1 is the only viral protein expressed on the surface of intact virions. It has the sole responsibility for the interaction with host cell receptors making it an attractive therapeutic mechanism for new agents. Variants with single point mutations within the major receptor loop of VP1 cannot bind to host cells [106,107]. These therapies would enable clinicians to effectively treat BK viraemia without compromising optimal immunosuppressive treatment.

1. Potravitug blocks the interaction of the viral capsid VP1 with the cell surface and, as a result, inhibits viral infection of cells. An initial phase 1 trial has demonstrated a safe dosing of 1000 mg given four times weekly across four weeks produces antibody levels sufficient to maintain VP1 receptor occupancy above 95% [108]. The SAFE Kidney II trial is a phase II and III placebo-controlled trial that has completed recruitment to assess for safety and efficacy of Potravitug [109]. Results of the phase II component of this trial have yet to be published.
2. MAU868 is a novel human IgG1 monoclonal high-affinity neutralising antibody against BKVAN. An initial phase I study has demonstrated that MAU868 is safe and well-tolerated [110]. The results of a phase II trial conducted in kidney transplant patients to examine the safety and efficacy of this drug are currently awaited [111].

### 8.3. Vaccination

BK vaccination, with a view to inducing high antibody levels of neutralising antibodies against BK virus, is of particular interest as a preventative measure for BK nephropathy, particularly in the pre-transplant cohort [81]. Recent consensus guidelines have highlighted this as an important area for future research and development [32]. It has been studied in macaque and mice models with the administration of virus-like particles which produced a broad neutralising response [112]. There is also recent research on developing a candidate mRNA vaccine against BK virus; however, there are no in vitro or in vivo studies in human cells. An effective BK vaccine would likely result in a significant decrease in peri-transplant BK nephropathy; however, it remains distant from clinical trials at present [113].

### 8.4. Novel Therapies and Targets

1. Agnoprotein has been postulated as a potential therapeutic drug target as it is an important small, positively charged protein with a pivotal role in the assembly, maturation and release of the BK virus. Initial studies have identified specific binding peptides against agnoprotein that may lead to targeted binding peptide therapeutics in the future and they may also have exciting potential in drug pharmacokinetics and diagnostic applications [114].
2. AIC468, a first-in-class antisense oligonucleotide designed to inhibit the splicing process of a viral mRNA encoding protein that is essential for BKV replication, is undergoing its first in-human phase 1 trial [115].

**Table 4:** A comparison of emerging therapies for BK virus.

Therapy	Mechanism	Trial Phase	Current Limitations
VST	Allogeneic T cells targeting BK virus.	Posoleucel completed phase 2 trial [96].	Expensive, labour-intensive, risk of cytokine storm.
	Immunomodulation via antigen specificity.		
ECP	Induces regulatory immune cells via UV-treated	No clinical trials.	Expensive, labour-intensive, lack of guidelines
	autologous leukocyte; reduces inflammation.	Used in case reports [104].	for use.
Potravitug	Neutralizing antibody blocks VP1–host receptor	Phase 1 completed [108].	Unclear duration of effect, no trial data in
	interaction, blocking viral entry.	Phase 2/3 underway [109].	kidney transplant cohort.
MAU 868	High-affinity IgG1 antibody targeting VP1, blocking	Phase 1 completed [110].	Unclear duration of effect, no trial data in
	viral entry.	Phase 2 underway [111].	kidney transplant cohort.
Vaccine	Induces neutralizing antibodies pre-transplant.	Pre-clinical studies [112,113].	Likely most efficacious in pre-transplant period.
		Evidence in animal models.	No clinical trials yet.
AIC468	Inhibits splicing of essential viral mRNA	Phase 1 underway [115].	First in-human trial, safety data awaited.
	to block replication.		

VST—viral-specific therapy, ECP—extracorporeal photophoresis.

## 9. Conclusions

BK virus remains an important cause of allograft failure, particularly given modern immunosuppressive techniques. Given the potentially serious complication of BKVAN, renal transplant programs have developed sophisticated screening programs that have improved BK virus detection. Important updates in screening include newer guidelines aiming to standardise screening and diagnosis internationally [32]. Screening and diagnosis of BK viraemia has become a rapidly changing field of research, with new urine-based testing including lateral flow assays, cell-free DNA and mRNA on the horizon. Despite significant advances, there remains scope for further research, including in the development of accurate risk prediction models. Unfortunately, there is not any available specific anti-viral treatment for BK viraemia. Current treatment strategies rely on immunosuppression reduction, which is associated with increased rates of graft rejection. However, there is a growing body of evidence for the use of cellular immunotherapy to reduce the risk of transplant rejection, and neutralising antibody treatments are actively being investigated in clinical trials.

## 10. Search Strategy

Articles selected for review were selected from PubMed, following a search of the title terminology, BK virus, BK viraemia and BK-associated nephropathy. Additional searches for specific subtitles were explored. Historical articles were reviewed to provide context to the paper, but an effort was made to focus on the best-quality evidence and papers published in the last five years.

## Figures and Tables

**Table 1:** BKPyV structure and functional components.

Component	Type	Function
VP1	Structural (major capsid protein)	Exposed on outside of the virion and essential for attachment to host cell receptors.
		Mediates capsid assembly and viral attachment to susceptible cells [5].
VP2 and VP3	Structural (minor capsid proteins)	VP1 binding region, a DNA binding region and a nuclear localisation signal.
		Not required for viral assembly/stability but removal reduces infectivity [6].
		VP3 leads to activation of ADP ribose polymerase, depleting ATP and cell death [7].
LTAg	Non-structural (regulatory)	Has a propensity to bind to p53 and protein Rb, commencing host cell cycle [8].
		Important roles in oncogenicity, viral replication and cellular recognition.
stAg	Non-structural (regulatory)	Modulates expression of LTAg, viral gene expression and DNA replication [9].
Agnoprotein	Non-structural (regulatory)	Viral channel protein that augments membrane permeability and
		facilitates viral particle release [10,11]. Deletion leads to failure to produce
		infectious progeny [12]. Furthermore, BK virus fails to release from host cells [13].
NCCR	Regulatory DNA	Contains the origin of replication and enhancer elements that modulate transcription.
		Mutations result in permissitivity, cell tropism and altered rates of replication [14].

VP—viral protein, LTAg—large T antigen, stAg—small T antigen, NCCR—non-coding control region.

**Table 2:** Risk factors for BK virus [32].

	BKPyV DNAemia	BKPyVAN
***Donor***		
Urinary BKPyV shedding	√	√
High donor antibody levels against BKPyV capsid protein VP1	√	
BKPyV genotypes different from the recipient	√	√
Certain donor BKPyV genotypes	√	√
***Recipient***		
Older recipient age	√	√
Male patient	√	√
Seronegative BKPyV if donor is positive	√	
Previous kidney transplantation	√	
HLA class I (absence of A2, B7, B8, B51, B44, B13, CW7)	√	
HLA class II (DR15)	√	
Interferon gamma gene rs2435061	√	
Younger paediatric recipient	√	
Obstructive uropathy as primary renal disease in paedatric recipient	√	
Low recipient neutralising antibody levels against donor BKPyV	√	√
***Transplant***		
Tacrolimus compared to cyclosporine	√	√
Lymphocyte-depleting agents	√	√
Acute rejection	√	√
Corticosteroids	√	√
mTor inhibitors	√	√
Ureteric stents	√	√
ABOi transplants	√	
BKPyV genome with rearranged NCCR		√

BKPyV—BK polyoma virus, VP—viral protein, HLA—human leukocyte antigen, ABOi—ABO incompatible, mTOR—mammalian target of rapamycin.

**Table 3:** Banff classification of polyoma virus.

Banff Class	Viral-Induced Tubular Change	Fibrosis
Class I	pvl 1	ci 0–1
Class II	pvl 1	ci 2–3
	pvl 2	ci 0–3
	pvl 3	ci 0–1
Class III	pvl 3	ci 2–3

pvl scoring is based on extent of tubules with viral change. pvl 1 ≤ 1% of tubules, pvl 2 > 1% to ≤ 10%, pvl 3 > 10%. ci scoring is based on interstitial fibrosis in cortex. ci0 0–5%, ci1 6–25%, ci2 26–50%, ci3 > 50%.
